# Intraosseous Temperature Predictors During Osteotomy with Helix GM (Titanium) and Zi Compact Drill Systems (Zirconium): A Linear Regression Study

**DOI:** 10.3390/dj14060354

**Published:** 2026-06-09

**Authors:** Sebastian Contreras, Daniel Alvitez-Temoche, Franco Mauricio, Fran Espinoza-Carhuancho, Oscar Sotomayor, Ivan Calderon-Cortez, Frank Mayta-Tovalino

**Affiliations:** 1 Faculty of Dentistry, Research, Innovation and Entrepreneurship Unit, Universidad Nacional Federico Villarreal, Lima 15088, Peru; scontra17@gmail.com (S.C.); dalvitezt@unfv.edu.pe (D.A.-T.); fmauricio@unfv.edu.pe (F.M.); osotomayor@unfv.edu.pe (O.S.); 2 Bibliometrics Evidence Evaluation and Systematic Reviews Group (BEERS) Human Medicine Career, Universidad Científica del Sur, Lima 15067, Peru; fransespinoza2.0@gmail.com; 3 EVIDENTIA Research Group, Universidad Nacional Mayor de San Marcos, Lima 15081, Peru; 4 Vicerrectorado de Investigación, Universidad San Ignacio de Loyola, Lima 15024, Peru

**Keywords:** drill osteotomy, in vitro, intraosseous temperature

## Abstract

**Background:** Excessive heat during implant osteotomy adversely affects bone healing and osseointegration. It is essential to carefully study the effects of drill systems, motors, and irrigation methods on intraosseous temperature. We aim to analyze the predictors of intraosseous temperature variation during osteotomy in bovine bone blocks using the Helix GM (Titanium) and Zi Compact (Zirconium) drill systems in conjunction with three different surgical motors and irrigation conditions using a linear regression model. **Materials and Methods:** An in vitro experimental study was conducted at the Periodontology and Oral Implantology Laboratory of the Universidad Nacional Federico Villarreal. A total of 120 bovine rib bone blocks (1.5 cm) were prepared using a standardized osteotomy protocol involving lance drills and ∅ 2 mm and ∅ 3 mm helical drills from the Zi and Helix GM compact kits (Neodent, Curitiba, Brazil). Irrigation was performed with chlorhexidine 0.12% + CPC 0.05% at ambient temperature (21 °C). Osteotomies were executed with two surgical motors (Coxo and Driller) at 1200 rpm and 35 Ncm torque. The intraosseous temperature was recorded in real time via a calibrated Fluke TiS55+ (Fluke, Everett, WA, USA) infrared thermographic camera and validated using a probe thermometer. Statistical analyses used Stata 17, applying descriptive measures, *t*-tests, and linear regression at 95% confidence for reliability. **Results:** Osteotomies without irrigation consistently resulted in slightly higher intraosseous temperatures. The Helix GM system, with the ∅ 3 mm drill and Driller motor, produced a final temperature of 29.3 °C ± 2.0. The Zi system with the lance drill and drill motor produced a maximum temperature of 32.7 °C ± 2.3. Irrigation was successful, and the elevated temperatures after irrigation were close to the surgical room temperature of 21–23 °C. Linear regression analysis showed that the drill motor produced a statistically significant decrease in temperature (−2.29 °C; 95% CI: −4.36 to −0.21; *p* = 0.031) while the lance drill with no additional irrigation produced a statistically significant increase in temperature (0.24 °C; 95% CI: 0.06 to 0.42; *p* = 0.009). **Conclusions:** The absence of irrigation during osteotomy significantly increased the intraosseous temperature, potentially compromising bone integrity. The use of irrigation, especially with the Driller motor, demonstrates a protective thermal effect.

## 1. Introduction

Dental implants, also known as endosseous implants, are considered a valuable contribution to dentistry, as they have revolutionized the way lost teeth are replaced [1,2], demonstrating long-term effectiveness and high success rates, which significantly improve the appearance, functionality, and quality of life of patients undergoing this treatment [3,4]. The success of dental implant treatment depends mainly on the biological phenomenon known as “osseointegration,” in which a direct structural and functional connection is formed between the surface of the dental implant and the patient’s bone [5]. This phenomenon, in turn, is the result of the interrelation of various factors, such as the macro- and microscopic characteristics of the implant surface, bone quality, surgical technique, and loading conditions [6,7].

The insertion of dental implants usually involves prior surgical procedures, such as osteotomy or bone drilling [8]. This procedure involves the use of surgical drills to make a hole in a specific site of the alveolar bone. However, this process inherently results in the generation of heat due to friction between the cutting edges of the drill and the adjacent bone [9]. A temperature of 47 °C for just one minute is enough to affect bone survival [10]. Therefore, to avoid bone necrosis and subsequent implant failure, it is recommended not to exceed this critical temperature threshold during bone drilling [11,12].

Currently, implant drills are manufactured from different materials, with stainless steel and zirconia being the most extensively studied. Each material presents unique mechanical and thermal properties that may influence surgical performance and biological responses, making their characterization essential for improving osteotomy techniques and ensuring predictable clinical outcomes. A problem that clinicians face when performing surgical drilling for dental implant placement is the generation of intraosseous heat, which is considered a high-risk factor for osteonecrosis [13].

There are several factors involved in the generation of heat during bone bed preparation, which may be directly related to the drill: material, shape, diameter, and wear; to the procedure itself: rotation speed, force applied, irrigation method, and irrigation temperature, among others; and to the patient’s bone [14,15]. Among all the factors mentioned above, the material of the bur, whether steel or zirconia, is one of the most likely to promote this increase in temperature [16,17,18,19].

This situation leads to an attempt to determine the temperature changes generated with different types of drills, whether metal (stainless steel) or ceramic (zirconia), which will help to decide the most optimal material to use in clinical practice to achieve a surgical preparation technique that is less traumatic for the bone and underlying tissues and consequently avoid premature failure of the dental implant. A distinctive aspect of this research lies in its focus on the thermal behavior of stainless steel and zirconia drills during osteotomy. By examining heat generation under controlled experimental conditions and applying linear regression to identify predictive factors, the study offers practical evidence that enriches clinical decision-making [10,11,12,13,14,15,16,17,18,19].

Therefore, this study aimed to analyze the predictors of intraosseous temperature during osteotomy procedures performed with the Helix GM (titanium) and Zi Compact (zirconia) drill systems, employing linear regression modeling to evaluate the independent contribution of drill geometry, motor performance, and irrigation conditions.

## 2. Methods

### 2.1. Study Design

The present investigation employed a controlled in vitro experimental design to assess the influence of different surgical drill systems and operative conditions on intraosseous temperature during implant osteotomy. Two commercially available compact surgical systems from the same manufacturer were evaluated: Helix GM and Zi Compact. Each system was tested in combination with two implant motors—C-Sailor Pro (Coxo, Foshan, China) and 600 Baby Brushless (Driller, São Paulo, Brazil)—under standardized rotational speed (1200 rpm) and torque (35 Ncm) conditions. Furthermore, osteotomies were performed using three sequential drill diameters (lance drill, ∅ 2 mm, and ∅ 3 mm) with and without external irrigation at room temperature. The study was conducted at the Periodontology and Oral Implantology Laboratory of the Faculty of Dentistry at the Universidad Nacional Federico Villarreal, Lima-Peru. The Checklist for Reporting in vitro Studies guidelines was used to report the findings [20].

### 2.2. Sample Size

**Figure 1:**
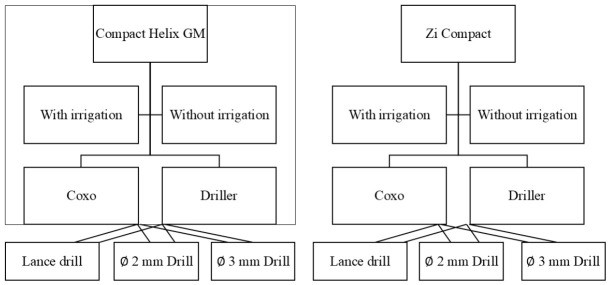
Allocation.

The sample consisted of 1.5 cm long blocks of bovine ribs obtained from the Camal La Colonial slaughterhouse (district and province of Callao). To calculate the sample size, a pilot study was conducted using the mean comparison formula, which consisted of n = 10 osteotomies for each study group, for a total of n = 120 bone preparations. All statistical analyses were conducted using Stata version 17.0. The comparison of means was performed using the appropriate formula to evaluate differences between experimental groups. Analyses were conducted with an alpha of 0.05 and a beta of 0.8. All experimental groups were assigned through a simple randomization process (Figure 1).

### 2.3. Selection Criteria

Only implant drills that were in a clean condition were included, belonging to two specific systems from the same manufacturer, with matching diameters and lengths, as well as 1.5 cm long bovine bone ribs, to ensure uniformity and control variables that could interfere with the results. In contrast, drills that were dirty, came from different manufacturers, or had previously undergone disinfection or sterilization processes were excluded to preserve the traceability of the initial conditions and avoid bias in the interpretation of the findings.

### 2.4. Obtaining and Preparing Bone Samples

Bovine bone ribs were obtained from the “Camal La Colonial” slaughterhouse (district and province of Callao) days before the test was performed. Once the bovine bone ribs were obtained, the meat was separated from the ribs and then cleaned to remove any soft tissue residue that may have remained on them. The ribs were then cut using a Model Al-116 Stainless Steel butcher’s saw (SAGAS, Lima, Peru) to obtain 120 bone blocks, each 1.5 cm long.

### 2.5. Selection of Surgical Drills

Commercially available surgical drills from the Zi compact surgical kit (Neodent, Brazil) and the Helix GM compact surgical kit (Neodent, Brazil) with similar diameter and length were used. Sequential drilling will be performed using the spear drill, the 2∅ helical drill, and the 3∅ helical bur from each surgical kit.

### 2.6. Surgical Protocol

**Figure 2:**
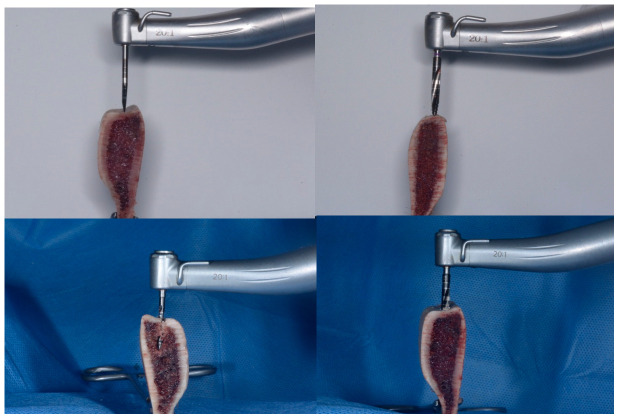
Drilling sequence. The drilling sequence for implant site preparation followed a standardized protocol. It began with the use of a lance drill to initiate osteotomy, ensuring precise positioning. Subsequently, a ∅ 2 mm drill was employed to establish the initial trajectory and depth of the site. Finally, a ∅ 3 mm drill was used to widen the osteotomy to the desired diameter, creating the appropriate conditions for implant placement.

Chlorhexidine (Perio-Aid, Dentaid, Barcelona, Spain) was applied at room temperature (21 °C) during the surgical drilling procedure. Through a random assignment process, bone blocks were allocated to each treatment combination to reduce potential bias in the order in which treatments were applied to the same experimental group (e.g., the first combination applied to each bone block). A total of 120 unique treatments were applied to each osteotomy block, providing a robust base from which to compare groups and enabling robust replication of thermal predictions. The drilling protocol was developed on 1.5-cm-long bovine bone ribs using a standardized sequence that included the use of a spear bur, a ∅ 2 mm helical bur, and a ∅ 3 mm helical drill. A constant speed of 1200 rpm, a controlled torque of 35 Ncm, and standardized continuous drilling without intermittent withdrawal were applied. To preserve the thermal conditions during osteotomy, external irrigation with 0.12% chlorhexidine + 0.05% CPC solution (Perio-Aid, Dentaid, Spain) at room temperature was used. The procedures were performed by an oral implantology specialist (Figure 2).

The implant drills used were manufactured by Neodent (Brazil) and were divided into two large groups according to the compact surgical system: Group 1, Helix GM; and Group 2, Zi. Each group was subdivided according to the presence or absence of irrigation, the type of surgical motor used—C-Sailor Pro (Coxo, China) and 600 Baby Brushless (Driller, Brazil)—and the diameter of the drill bit used (lance, ∅ 2 mm, ∅ 3 mm), systematically covering all possible combinations.

To ensure standardization of the experimental protocol, the ribs were previously sectioned with a stainless-steel AL-116 meat and bone saw (SAGAS, Lima, Peru). Thermal measurements were performed in real time using a Fluke TiS55+ infrared camera (Fluke, Everett, WA, USA), calibrated by a specialized engineer, and supplemented with a wired probe thermometer (Taylor, Oak Brook, IL, USA) to validate the obtained records.

To help minimize the risk of bias when assigning bovine bone samples into experimental groups, block randomization was used. The following environmental factors were kept track of so that fluctuations in ambient temperature and relative humidity did not distort experimental results: bovine bone density was calibrated prior to drilling; each operator received training in the same manner to ensure uniformity; the number of times a drill bit was used and sharpened was recorded; and all specimens were kept in hydrated conditions to maintain tissue qualities needed for uniformity and minimize any effects attributable to dehydration.

### 2.7. Intraosseous Temperature Measurement

**Figure 3:**
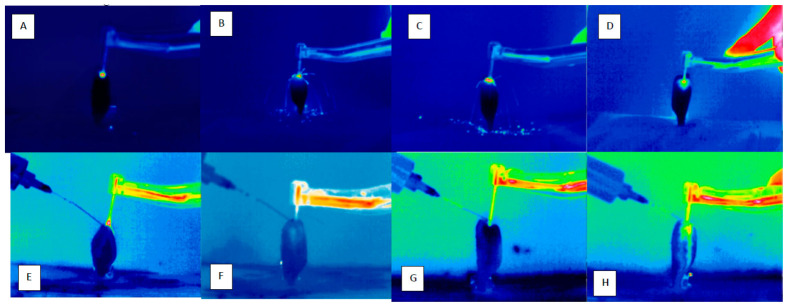
Infrared heat generated (°C) by drills for Helix GM (Titanium) and Zi Compact drill systems (Zirconium)with and without irrigation. Intraosseous temperature was recorded in real time using a calibrated Fluke TiS55+ infrared thermography camera positioned 20 cm from the bone blocks, ensuring standardized high-resolution thermal imaging and precise interpretation. (**A**–**D**) illustrate the drilling sequence without irrigation, whereas (**E**–**H**) depict the sequence performed using irrigation.

The intraosseous temperature was measured using real-time infrared thermography with a Fluke TiS55+ thermal imaging camera (Fluke, Everett, WA, USA), which was properly calibrated by a specialist engineer before the experimental protocol began. The camera was placed at a focal distance of 20 cm from the bovine bone blocks, allowing for the standardized capture of high-quality thermal images exceeding the RESNET resolution of 220 × 165. Its flexible manual focus enabled precise close-ups of up to 15 cm, with a depth of field of 353:1. In addition, the five IR-Fusion^®^ mode presets were used to improve thermal interpretation, and a 3.5-inch (320 × 240 px) screen was used, which provided 33% more viewing area than conventional screens (Figure 3).

### 2.8. Data Analysis

For the statistical processing of the data obtained in this in vitro experimental study, Stata version 17.0 for Windows 11 software will be used. Descriptive statistics included the grouping of data into structured tables to facilitate analysis, using measures of central tendency, such as the mean, as well as measures of dispersion, such as the standard deviation. The normality of the data distribution was assessed using the Shapiro–Wilk test. For inferential analysis, parametric tests, such as the Student’s *t*-test, were applied to compare the means of the different experimental groups, and linear regression was used to evaluate possible associations between the previously defined dependent and independent variables. All analyses will be conducted at a 95% confidence level, in accordance with the methodological standards required in health science research, thus ensuring the robustness and reliability of the findings.

## 3. Results

### 3.1. Osteotomies with the Compact Helix GM Surgical System

The results obtained using the Helix GM Compact Surgical System showed significant differences in the intraosseous temperature between the osteotomies performed with and without irrigation. Under irrigation conditions, the recorded averages were 21.0 °C ± 0.5 (∅ 2 mm, Coxo motor) and 23.6 °C ± 1.2 (∅ 2 mm, Driller motor), while in the absence of irrigation, higher temperatures were observed, such as 24.8 °C ± 1.1 (∅ 2 mm, Coxo motor), 25.7 °C ± 1.2 (∅ 3 mm, Coxo motor), and up to 29.3 °C ± 2.0 (∅ 3 mm, Driller motor). Student’s *t*-test confirmed the significance of these differences, highlighting the positive effect of irrigation on thermal control during osteotomy, which helps preserve bone tissue integrity in implant procedures (Table 1).

### 3.2. Osteotomies Performed Using the Zi Compact Surgical System

The findings obtained using the Zi Compact Surgical System revealed significant differences in the intraosseous temperature values between the drilling conditions with and without irrigation. Under irrigation, lower average temperatures were recorded, such as 21.5 °C ± 0.3 (∅ 2 mm, Coxo motor) and 21.8 °C ± 0.7 (∅ 3 mm, Coxo motor), as well as 22.6 °C ± 0.9 (∅ 2 mm, Driller motor) and 22.8 °C ± 2.1 (∅ 3 mm, Driller motor). In the absence of irrigation, the values were considerably higher, reaching 24.5 °C ± 1.7 (∅ 2 mm, Coxo motor), 26.2 °C ± 1.6 (∅ 3 mm, Coxo motor), and up to 30.9 °C ± 2.5 (∅ 3 mm, Driller motor), with a maximum recorded temperature of 32.7 °C ± 2.3 for the spear drill with Driller motor. As with the previous implant system, statistical significance was evident between these differences (Table 2).

### 3.3. Predictors of Temperature

The exploratory linear regression model applied to the intraosseous temperature recorded during drilling with a ∅ 3 mm drill under irrigation demonstrated the following: When considering surgical systems, for each unit of variation, the use of the Zi compact system was associated with a temperature drop of 0.39 °C (95% CI: −1.38 to 0.59). The use of the Driller motor was associated with a statistically significant drop in temperature of 2.29 °C (95% CI: −4.36 to −0.21), indicating a significant thermal protective effect.

Regarding the type of drill and irrigation condition, the use of the irrigation drill barely changed the average temperature (−0.02 °C; 95% CI: −0.36 to 0.31), without reaching statistical significance. In contrast, the drills without irrigation increased the temperature by 0.24 °C (95% CI: 0.06–0.42), which was statistically significant, suggesting that the absence of irrigation generates a temperature increase even in the initial stages of drilling.

The use of the ∅ 2 mm drill with irrigation showed a non-significant increase of 0.10 °C (95% CI: −0.33 to 0.55), whereas the same drill without irrigation showed a variation of 0.09 °C (95% CI: −0.21 to 0.41), also not significant, although with a tendency toward thermal increase. Finally, the use of the ∅ 3 mm drill without irrigation did not change the temperature (0.00 °C; 95% CI: −0.21 to 0.21), reflecting the absence of a relevant effect in this specific phase. The model provided an R^2^ of 0.22 (i.e., 0.08 after adjustment for predictors not expected to affect the dependent values), meaning that around 22% of the raw temperature value was explained by the predictors. The model was ‘not significant’ (Prob > F = 0.1565) although some components, such as the ‘baby drill motor’ (*p* = 0.031) and the lance drill not irrigated (*p* = 0.009), did have a significant effect. Assumptions of linearity, independence, and homoscedasticity were made, and p-values were stated for each coefficient of the regression so that the reader can appreciate the veracity of the results (Table 3).

## 4. Discussion

It is important to highlight that we compared two systems of surgical drills (Helix GM and Zi Compact—Neodent); we did not compare implants of different materials. The drills used were from a commercial brand and did not differ in composition, other than the system from which they came; in the same way, we did not compare zirconium and titanium implants. The greatest takeaway from this study lies in the thermal behavior of drill systems, motor types, and irrigation conditions; thus, we have modified the title and objectives to not misconstrue the focus of this study. Student’s *t*-test was used to examine differences in mean temperatures between conditions, allowing for bivariate differences to be identified under parametric conditions. However, as heat generation is a function of multiple factors acting simultaneously, univariate comparison was inadequate. Therefore, linear regression was used to model temperature as a continuous variable and included the milling system, motor, diameter, and irrigation to estimate the adjusted effect. This combination approach first identified simple differences and then assessed the independent contribution of the categorical predictors, thereby improving the inferential strength of the analysis.

This research analyzed and discussed various variables that may have some impact on the generation of intraosseous heat during the preparation of the implant bed, such as the surgical kit, the implant motor, the presence or absence of irrigation, and the diameter of the drill (spear drill, ∅ 2 mm and ∅ 3 mm), which allowed for a much broader understanding of the possible variables strongly related to the increase in bone tissue temperature [21].

In the Compact Helix GM surgical kit, the instrument that demonstrated the most significant average intraosseous temperature rise was the 3∅ mm drill when utilized with the drill motor and without irrigation (29.3 °C). The instrument that demonstrated the least average temperature generation during preparation was the 2∅ mm drill bit, which was used with the Coxo Motor and chlorhexidine irrigation (21.0 °C). In the Compact Zi surgical kit, the bur that created the most elevated temperature increase in the bone was the Lance drill, with no irrigation used (32.7 °C), used with the Driller Motor. The implant drill that produced the least average temperature increase in osteotomy was the 2∅ mm drill when used with the Coxo Motor and chlorhexidine irrigation (21.5 °C). In summary, it was demonstrated that when utilizing the Coxo Motor with chlorhexidine irrigation, the 2∅ mm drill bit created the least intraosseous temperature increases, irrespective of the surgical kit (Compact Helix GM or Compact Zi) being utilized.

Also, our results suggest that intraosseous temperature differences are better described by mechanisms of heat generation and dissipation in the intraosseous environment than by the drill system label. The high level of protection afforded by the Baby Drill motor (−2.29 °C; *p* = 0.031) is likely due to both high torque efficiency and acceleration of cutting energy being ‘smoother’, which results in less production of frictional heat at the drill–bone interface. The lowest recorded cooling (0.24 °C; *p* = 0.009) of the lance drill without irrigation suggests that frictional force coupled with limited cutting efficiency predisposes the ‘temperature chair’ to higher accumulation of heat in the absence of a coolant. The results indicate that irrigation acts as a thermal regulator, dissipating heat away from the bone regardless of drill diameter or system, and that the degree of transferring heat is regulated by motor performance and drill geometry.

On the other hand, when using the Driller Motor without irrigation, the instruments that produced the highest temperature increases in bone tissue were the Lance and 3∅ drills for both surgical kits used in the research (Compact Helix GM or Compact Zi). In addition, the use of irrigation during surgical preparations showed the lowest average intraosseous temperature values regardless of the surgical system, implant motor, and drill used, with the exception of the Lance drill of the Compact Helix GM and Zi systems with the Coxo motor, where their non-irrigated counterparts surprisingly generated a lower average temperature increase (22.0 °C and 21.6 °C, respectively). This demonstrates the importance of using coolants during osteotomies for implants, as they significantly reduce the temperature generated.

In addition, when comparing the temperature generated when using the same drill diameter, both without irrigation but with different implant motors, it was found that, regardless of the surgical kit used, when these were used with the Coxo Motor, the temperature of each drill (Lance, 2∅, and 3∅ mm) was lower than that of their counterparts without irrigation with the Driller Motor. On the other hand, when comparing the temperature generated using the same drill diameter belonging to the Compact Zi Surgical Kit, both with chlorhexidine irrigation, but with different surgical motors, it was revealed that all drills (Lance, 2∅, and 3∅ mm) generated lower intraosseous temperature increases when used with the Coxo Motor than when used with the Driller Motor. However, this comparison did not hold true when using the Compact Helix GM Surgical Kit. Thus, the surgical motor used for implant site preparation is also an important variable to consider reducing the temperature values generated in the bone.

One of the most notable challenges faced by clinicians during implant site preparation is the inherent generation of heat in the bone and the possible thermal damage that this can cause. The heat generated during this surgical procedure is mainly linked to three major factors: (a) those related to the drilling procedure itself, including irrigation, drilling technique, rotation speed, and drilling force, among other [12]; (b) those related to the characteristics of the drill; and (c) those related to the characteristics of the patient’s bone [11].

It has been reported that when the intraosseous temperature generated during surgical drilling increases 10 °C above the body temperature for 1 min or more, reaching 47 °C, bone tissue necrosis (osteonecrosis) occurs, resulting in osseointegration failure. Having explained this, maintaining the intraosseous temperature below this threshold is critical for successful osseointegration [22,23].

One of the most relevant contributions of this research lies in the importance of continuous cooling during surgical drilling procedures, as this significantly reduces the intraosseous temperature generated, which is therefore considered one of the main factors to be considered to minimize the inherent temperature rise as much as possible and thus optimize the osseointegration of the future dental implant.

Paeßens et al. determined that the presence or absence of irrigation is a factor that contributes significantly to the increase or decrease in intraosseous temperature, since the application of saline irrigation at room temperature during osteotomies generated lower temperatures than their counterparts without irrigation, which exceeded the critical temperature limit for different drill diameters (2.4, 3.2/3.6, and 3.8/4.2 mm) and rotation speeds (200, 600, and 1000 rpm), concluding that osteotomies in oral implantology should not be performed without cooling, even at low rotation speeds, to avoid or prevent heat-induced bone damage [24].

Barriga-Yauri et al. [21], when evaluating the influence of irrigation, drill diameter, implant motor, and surgical system on intraosseous temperature generation, determined that, in agreement with the previous study, the presence of irrigation is extremely necessary and indispensable for reducing the temperature values generated in bone tissue during osteotomies, regardless of the surgical system, implant motor, and drill diameter used, results that converge with those of the present investigation.

Şarkbay et al. [25] evaluated the temperature changes caused by the drills of five different surgical systems, considering the presence or absence of cooling (isotonic solution), rotation speed (120 and 1200 rpm), and age of the drill (new and used). The results revealed a non-significant difference in the heat generated with the five drill systems used, which coincides with the results of the current study. In addition, temperature changes were greater when the drills were used without irrigation, regardless of the system, rotation speed, and age of the drill, which again highlights its importance during these surgical procedures. On the other hand, the study by Guler et al., which compared five surgical drill systems, including the variables of drilling speed (150, 250, and 400 rpm), drill diameter, and no irrigation, showed variable results in terms of maximum temperature depending on the drill diameter, which is consistent with the results of this research [26].

Future research in this area should move beyond isolated variables and embrace a more integrative perspective, examining how drill geometry, motor efficiency, and irrigation techniques interact under realistic clinical conditions. Incorporating advanced thermographic monitoring, larger sample sizes, and in vivo models will allow for a deeper understanding of heat generation and dissipation. Such approaches may inspire innovative cooling strategies, ultimately guiding safer implant site preparation and fostering more predictable osseointegration outcomes.

## 5. Suggestions and Limitations

This study presents certain limitations that should be acknowledged. The absence of a universal testing machine restricted the standardization of drilling force and feed/retraction speed, which were instead controlled manually by an experienced implantologist. Future investigations would benefit from mechanical standardization to reduce variability. Additionally, the use of room-temperature irrigants may have underestimated the cooling potential of chilled solutions. Finally, as an in vitro design, the use of bovine bone blocks—lacking blood flow and physiological regulation—limits direct extrapolation to clinical scenarios. Nevertheless, these controlled conditions provide valuable insights into intraosseous heat dynamics and strategies for protecting bone tissue during osteotomy [27,28].

A limitation of this study was the use of chlorhexidine at room temperature as an irrigant instead of saline, which is the most common fluid in clinical implant procedures. Chlorhexidine was selected for consistency and its documented antimicrobial properties in laboratory simulations; however, this choice was empirical and not intended as a clinical recommendation. Although previous evidence suggests that chlorhexidine does not alter heat conduction in bone compared to saline, the findings should be strictly interpreted within the controlled experimental context.

While it has been clearly shown that the cooling effect of any irrigation on osteotomy, particularly involving a drill, is present, the literature does not describe a situation where the interaction between some design of the drill system, motor, diameter of drill, and irrigation is ever looked at in the same controlled analytic model. An often-used drill system design is considered in isolation, limiting a multilinear multivariate interpretation. This study intends to address those shortcomings in the literature by applying factorial design as an original corrective step to employ regression analysis to elucidate the effect of the operative variables in a more straightforward way.

Finally, the results of this study were obtained from a drilling protocol performed by a single highly trained and skilled operator who executed all drilling procedures under manual control. Thus, there is an inherent limitation to the results as there may be uncontrolled variation in many variables (drilling pressure, feed rate, drill angulation, operator fatigue, etc.) due to the nature of the study design. Although there was no way to accurately quantify these potential sources of variation, they could affect the thermal values measured during osteotomy. Nevertheless, every effort was undertaken to minimize this bias by using standardized protocols, specifically: rotational speed, torque, drill sequence, irrigant temperature, and a carefully controlled randomization system to distribute bone blocks among experimental groups. Although our statistical analysis did not account for operator-related variation, the very tight control of the experimental conditions and balanced replication across experimental groups served to augment the internal validity of our study and provided a basis for making reasonable conclusions regarding the results of the exploratory study.

## 6. Conclusions

Within the limitations of this in vitro study, this research indicates that intraosseous heating caused by drilling during an osteotomy is affected using irrigation, drill bit size, and motor type. Compared to the non-irrigated conditions, all non-irrigated conditions showed higher intraosseous temperatures than irrigated conditions, with increases of up to 7–10 °C, depending on the drill bit and motor combination used. Larger diameter drill bits and the use of the “Driller” motor produced the greatest increase compared to other drill/motor combinations. All irrigated conditions showed similar intraosseous temperature readings within a relative range of 21–23 °C for the systems evaluated. Analysis of these data, supported by an exploratory linear regression model, indicated that the use of irrigation with the “Driller” motor resulted in a statistically significant relative reduction in intraosseous temperature, whereas the absence of irrigation produced significant and measurable increases in intraosseous temperature from the initial assessment points in this experiment. The results of these experiments support the use of an external irrigation solution applied at room temperature to minimize thermal effects during osteotomy, thereby improving the surgical procedure by providing optimal conditions for bone tissue healing and regeneration.

## Figures and Tables

**Table 1:** Osteotomies performed using the Compact Helix GM Surgical System.

				With Irrigation				Without Irrigation			
System	Motor Type	Drill	n	Mean	SD	*p* *	n	Mean	SD	*p* *	*p* **
Compact Helix GM	Coxo	Lance Drill	10	22.7	0.4	0.869	10	22.0	0.5	0.000	0.004
		∅ 2 mm Drill	10	21.0	0.5	0.570	10	24.8	1.1	0.152	0.000
		∅ 3 mm Drill	10	22.6	0.7	0.775	10	25.7	1.2	0.348	0.000
	Driller	∅ Lance Drill	10	21.7	0.3	0.841	10	25.0	1.2	0.931	0.000
		∅ 2 mm Drill	10	23.6	1.2	0.868	10	26.3	1.3	0.000	0.000
		∅ 3 mm Drill	10	21.1	0.4	0.544	10	29.3	2.0	0.943	0.000

Values were expressed in °C (Celsius degrees). *p* *: Shapiro–Wilk normality test. *p* **: Student’s *t*-test. The data showed homogeneity of variances *p* > 0.05.

**Table 2:** Osteotomies performed using the Zi Compact Surgical System.

				With Irrigation				Without Irrigation			
System	Motor Type	Drill	n	Mean	SD	*p* *	n	Mean	SD	*p* *	*p* **
Zi Compact	Coxo	Lance Drill	10	22.0	0.4	0.846	10	21.6	0.6	0.186	0.094
		∅ 2 mm Drill	10	21.5	0.3	0.036	10	24.5	1.7	0.450	0.000
		∅ 3 mm Drill	10	21.8	0.7	0.960	10	26.2	1.6	0.231	0.000
	Driller	Lance Drill	10	22.8	2.3	0.011	10	32.7	2.3	0.041	0.000
		∅ 2 mm Drill	10	22.6	0.9	0.666	10	26.2	0.6	0.444	0.000
		∅ 3 mm Drill	10	22.8	2.1	0.000	10	30.9	2.5	0.133	0.000

Values were expressed in °C (Celsius degrees). *p* *: Shapiro–Wilk normality test. *p* **: Student’s *t*-test. The data showed homogeneity of variances *p* > 0.05.

**Table 3:** Linear regression analysis to determine the predictors of Temperature with ∅ 3 mm drill with irrigation.

		Temperature (°C) with ∅ 3 mm Drill with Irrigation	
Variables		β	
	Coefficient	*p*	CI 95%
Ref. Zi Compact System	−0.39	0.423	−1.38 to 0.59
Ref. Motor Baby Drill	−2.29	0.031	−4.36 to −0.21
Lance Drill with Irrigation	−0.02	0.893	−0.36 to 0.31
Lance Drill without Irrigation	0.24	0.009	0.06 to 0.42
∅ 2 mm Drill with Irrigation	0.10	0.625	−0.33 to 0.55
∅ 2 mm Drill without Irrigation	0.09	0.533	−0.21 to 0.41
∅ 3 mm Drill without Irrigation	0.00	0.993	−0.21 to 0.21

## Data Availability

The data presented in this study are available on request from the corresponding author.
